# Self-Injury in Adolescence Is Associated with Greater Behavioral Risk Avoidance, Not Risk-Taking

**DOI:** 10.3390/jcm11051288

**Published:** 2022-02-26

**Authors:** Alina K. Dillahunt, Daniel A. Feldman, Leah R. Thomas, Brian W. Farstead, Summer B. Frandsen, Somi Lee, Myah Pazdera, Jennica Galloway, Katie L. Bessette, Henrietta Roberts, Sheila E. Crowell, Edward R. Watkins, Scott A. Langenecker, Melinda Westlund Schreiner

**Affiliations:** 1 Department of Psychiatry, Huntsman Mental Health Institute, University of Utah, Salt Lake City, UT 84108, USA; alina.dillahunt@hsc.utah.edu (A.K.D.); daniel.a.feldman@utah.edu (D.A.F.); lthomas@sa.utah.edu (L.R.T.); brian.farstead@utah.edu (B.W.F.); summer.frandsen@utah.edu (S.B.F.); somi.lee@utah.edu (S.L.); myah.pazdera@utah.edu (M.P.); jennica.galloway@utah.edu (J.G.); kbessette@mednet.ucla.edu (K.L.B.); sheila.crowell@psych.utah.edu (S.E.C.); s.langenecker@hsc.utah.edu (S.A.L.); 2 Department of Psychology, University of Utah, Salt Lake City, UT 84112, USA; 3 Department of Psychology, University of Illinois at Chicago, Chicago, IL 60607, USA; 4 Semel Institute for Neuroscience and Human Behavior, University of California Los Angeles, Los Angeles, CA 90024, USA; 5 Department of Psychology, University of Exeter, Exeter EX4 4PY, UK; h.roberts@exeter.ac.uk (H.R.); e.r.watkins@exeter.ac.uk (E.R.W.); 6 Department of Obstetrics and Gynecology, University of Utah, Salt Lake City, UT 84112, USA

**Keywords:** adolescence, self-injury, impulsivity, risk-taking, depression

## Abstract

Strategies to link impulsivity and self-injurious behaviors (SIBs) show highly variable results, and may differ depending on the impulsivity measure used. To better understand this lack of consistency, we investigated correlations between self-report and behavioral impulsivity, inhibitory control, SIBs, and rumination. We included participants aged 13–17 years with either current or remitted psychopathology who have (*n* = 31) and who do not have (*n* = 14) a history of SIBs. Participants completed self-report measures of impulsivity, the Rumination Responsiveness Scale (RRS), and two behavioral measures of impulsivity: the Balloon Analogue Risk Task (BART) and Parametric Go/No-Go (PGNG). Lifetime SIBs were positively associated with self-reported impulsivity, specifically positive and negative urgency. However, individuals with greater lifetime SIBs demonstrated greater risk aversion (lower impulsivity) as measured by the BART, whereas there was no relation between lifetime SIBs and PGNG performance. There was no relation between rumination and behavioral impulsivity, although greater rumination was associated with higher negative urgency. Future research examining the role of SIBs in the context of active versus remitted psychopathology is warranted. Because most adolescents were remitted from major depressive disorder at the time of study, follow-up studies can determine if lower risk-taking may aid individuals with more prior SIBs to achieve and maintain a remitted state.

## 1. Introduction

Suicide is the second leading cause of death for adolescents, with nonsuicidal self-injury (NSSI), rumination, and prior suicide attempts being identified as risk factors for future suicide attempts and deaths [1,2,3] (CDC). In order to refine preventative interventions that target at-risk individuals, we must establish more appropriate identification and measurement strategies of these risk factors. When researching at-risk individuals, a common focus is impulsivity as it is frequently considered a risk factor for self-injurious behaviors (SIBs). While some studies have supported an association between impulsivity and SIBs, other studies have failed to show this relation [4]. This highlights the variability in the conceptualization and measurement of impulsivity.

Impulsivity is a multifaceted construct that includes urgency, sensation seeking, inattentiveness, lack of perseverance and premeditation, disinhibition, and distractibility [5,6,7,8]. Impulsivity can be measured in several ways and is often clinically assessed using self-report measures. Common self-report measures include the Urgency, Premeditation (lack of), Perseverance (lack of), Sensation Seeking, Positive Urgency, and Impulsive Behaviors Scale [8] (UPPS-P) and the Barratt Impulsivity Scale-11 [9,10] (BIS-11) which measures motor, attentional, and nonplanning impulsivity.

Studies focused on self-report measures of impulsivity have highlighted the relation between impulsivity and SIBs. Higher ratings of self-report impulsivity, as opposed to behavioral measures of impulsivity, have been linked to more severe NSSI in adolescents [11]. Similarly, in a recent literature review with adolescents and young adults, self-reported impulsivity, especially the facet of urgency as measured by the UPPS, was positively associated with lifetime SIBs [12]. Also in this review, other self-report measures of impulsivity, such as the BIS-11, had a less clear association, often not linking to NSSI. In contrast, a more recent study did find a meaningful link between higher BIS-11 scores and greater NSSI [13]. With regard to both self-report and behavioral impulsivity, other studies of adolescents have not consistently found a relationship between self-injury and impulsivity [14].

Self-report measures inherently require insight into one’s own behavior. As such, self-report measures may be inaccurate or have a response bias, which could be a reason for this inconsistent link between self-report impulsivity and SIBs [15,16]. Therefore, it is important to investigate the SIB and impulsivity link using a less-biased measure such as lab-based behavioral impulsivity measures. Behavioral measures of impulsivity, while less frequently used in clinical settings, are often used in research. Lab-based impulsivity tasks such as go-no-go (GNG) paradigms measure impulsivity in information processing, inhibitory control, and motor restraint, a distinctly different form of impulsivity than self-reported impulsivity [17,18]. Other common paradigms measure response inhibition, decision making, and risk-taking behavior [19,20,21]. Behavioral impulsivity tasks commonly used in SIB research include the Eriksen Flanker task and Stop Signal Task, which measures response inhibition, and the Go/No-Go Task, which measures preemptive inhibitory control. While these tasks limit the effect of bias present in self-report data, all three of these lab-based tasks have shown little to no relation to either SIBs or self-report measures of impulsivity [17,22,23]. Finding objective measures related to SITBs may help identify individuals who are self-harming, but who are not willing to disclose, and could thus aid in screening.

Given the lack of relationship with SIBs or self-reported impulsivity, different behavioral-based approaches to capture characteristics of impulsivity are needed to assess risk more accurately. In particular, the behavioral impulsivity measures used to date in SIB research are typically focused on measuring the ability to inhibit motor response in a nonemotional context. Motor inhibition is a distinctly different facet of impulsivity compared to those aspects of impulsivity captured in self-report, namely inhibition and self-regulation of impulsive behavior in everyday life [24]. Moreover, lab-based impulsivity and inhibition tasks usually use nonemotional stimuli in a controlled environment, whereas self-report measures of impulsivity involve recall of potentially highly-emotional life experiences.

The inclusion of more emotion-related content may help enhance behavioral measures of impulsivity. In particular, adolescents with higher lifetime SIBs show reduced ability to inhibit responses to negatively-valanced items in the Stop Signal Task [25]. Behavioral measures may also benefit from examining other aspects of impulsivity. For instance, the Balloon Analogue Risk Task (BART) is a measure of risk-taking that is associated with impulsive choice (as opposed to motor inhibition), behavioral constraint, and risk behaviors [26,27,28,29]. The BART requires participants to earn as much money (or points) as possible by pumping a simulated balloon without having it pop. It can be conceptualized as an emotional-laden risk task where noises are used to indicate wins and losses, and there is a constant reminder of points on the screen. The BART performance has been shown to highly correlate with self-reported impulsivity in one study [27], though only weakly in others [30,31]. Furthermore, a handful of studies have linked NSSI with the BART performance among adults [32] and young adolescents [33] (10–13-year-olds).

A tertiary factor we consider useful in conceptualizing behavioral impulsivity and SIBs is rumination. Rumination, the habit of thinking about past events in a passive and abstract way—often described as getting stuck in a pattern of thinking—can be linked to SIBs [34]. Rumination can lead to increases in negative affect that then leads to more rumination, starting an emotional cascade. To escape from this negative cascade, individuals with a higher tendency toward impulsivity will sometimes engage in SIBs and other risk behaviors in an effort to regulate emotions [35,36,37]. Likewise, rumination has been linked to suicidal ideation in adults [38,39] and NSSI in adolescents [40]. The tendency to ruminate may lay the groundwork for negative affective states that contribute to impulsivity within emotional contexts and may be pronounced in individuals with SIBs.

In the current study, we investigated the relations between self-report impulsivity, risk-taking impulsivity measured using the BART, rumination, and SIBs. We included inhibitory control impulsivity measured with the Parametric Go/No-Go (PGNG) in our analyses to confirm prior results suggesting a weak correlation to self-report impulsivity and SIBs. Assessing differences and relationships between PGNG and BART performance will also lend insight to whether the BART is truly capturing risk-taking behaviors, or pushing to gain points, and avoidance of risk, or stopping trials to avoid balloon popping and loss of points. We believe that self-report measures capture a participant’s theoretical understanding of their own impulsivity through reporting factors, such as urgency, inattentiveness, and distractibility. Conversely, behavioral measures give the participant a goal and rules to follow. Facets of impulsivity, such as risk-taking or inhibitory control, are then directly measured through motor responses. Therefore, we hypothesized that there may be a weak association if any between self-report impulsivity and our behavioral measure of motor inhibition. Given its more well-established link in the literature [12], we anticipate that we will see a positive relationship between lifetime SIBs and the facet of urgency within the self-report measures of impulsivity. Based on existing literature, we also predict a positive relation between SIBs and BART risk-taking performance [33]. Finally, we explore the relationship of rumination as it relates to SIBs and impulsivity, as it may contribute to an increased risk of maladaptive coping behaviors.

## 2. Method

### 2.1. Participants

Adolescent participants aged 13–17 years were recruited from the Salt Lake City, UT area through radio advertisements, social media posts, and an electronic data warehouse. Participants were included in three studies, provided they had complete and usable data. Inclusion and exclusion criteria for each study are shown in Table 1.

Prior to enrollment, written consent and assent were obtained from adolescents and a legal guardian. All three studies were approved by the institutional review board at the University of Utah.

### 2.2. Measures

#### 2.2.1. Clinical Assessment

The Schedule for Affective Disorders and Schizophrenia for School Aged Children–Lifetime Version [41] (KSADS-PL) was used to determine prior or current mental health diagnoses for studies 1 and 2. The Mini-International Neuropsychiatric Interview for Children and Adolescents (MINI-Kid) was used for study 3 [42]. The Lifetime Suicide Attempt Self-Injury Count [43] (LSASI) was used to collect more in-depth information about SITBs, including number of lifetime self-injurious behaviors, method and severity of self-injuries for studies 1 and 2. For study 3, SITBs were assessed using the Self-Injurious Thoughts and Behaviors Interview [44] (SITBI). Across these measures, we used the MDD status (active vs. remission) and nonsuicidal and suicidal self-injuries lifetime count.

#### 2.2.2. Self-Report

Impulsivity. Studies 1 and 2 collected self-report impulsivity data using the UPPS-P, which consists of five impulsivity factors: Negative and positive urgency, lack of perseverance, lack of premeditation, sensation seeking, and impulsive behaviors [8]. Negative and positive urgency measure mood-based feelings of urgency with items, such as “When I feel bad, I often do things I later regret in order to make myself feel better now” and “I tend to act without thinking when I am very, very happy.” Lack of perseverance has items, such as “I like to see things through to the end”, while lack of premeditation has items, such as “I tend to blurt out things without thinking.” Sensation seeking has items, such as “I like new, thrilling things to happen.” Study 3 collected self-report impulsivity using the Three Factor Impulsivity Index (TFI), which borrows items from other established impulsivity measures including the UPPS-P [5]. Due to the small sample size of individuals (*n* = 5) who completed the TFI as opposed to the UPPS-P, we only included participants who completed the UPPS-P in our evaluation of self-reported impulsivity associated with SIBs.

Rumination. The Rumination Responsiveness Scale [45] (RRS) is a 22-item survey where participants rate each item using a 4-point Likert scale (1 = never to 4 = always). The RRS measure has three subscales that measure pathological brooding about causes and meanings of troubles, reflective pondering, and rumination about depressive symptoms.

#### 2.2.3. Neuropsychological Testing

Participants completed a (1–2 h) battery of neuropsychological tests, either in person or virtually. Brief breaks were allowed between tasks to prevent fatigue. If conducted virtually, participants were in a quiet room with their own computer and connected to a team member via video conference during the testing. Information on internet speed and browser were recorded. If conducted in person, participants were in a quiet room on a lab computer with a team member present during the testing.

Balloon Analogue Risk Task. The Balloon Analogue Risk Task [27] consists of a practice trial followed by 30 experimental trials. The participant sees a small balloon on the screen, and every time they press the ‘p’ key it pumps the balloon bigger, and they earn 10 points. The balloon is set to pop at random, and the participants are told that the balloon can pop at any time, and if it pops, they lose every point they earned for that trial. Therefore, participants must decide for each trial whether to continue pumping to earn more points but risk losing them all if the balloon pops or whether to push ‘s’ to stop for that round and collect the points toward their cumulative total. Important measures from this task include mean number of pumps for every balloon that did (PMP) and did not pop (NPMP) and number of points lost through exploding balloon trials.

Parametric Go/No-Go. The Parametric Go/No-Go (PGNG) computer task measures inhibitory control, attention, set shifting, and processing speed [46]. The computerized task consists of a series of letters or shapes that are presented for 500 ms each with no interval in between, and the participant must respond or inhibit a response as quickly and accurately as possible by clicking a set keyboard key. During the Go level, participants respond to two presented targets (i.e., “circle” or “diamond” for shapes, “r” or “s” for letters) every time they appear (e.g., all targets rule). During the No-Go level, participants respond to two targets (i.e., “circle” or “diamond”) every time they appear without repetition (e.g., nonrepeating rule), meaning they must inhibit a response if the target repeats. In the final difficulty levels, participants follow the same rules but this time with three items in the target set (i.e., “circle”, “diamond”, or “triangle”) to increase difficulty. Accuracy is measured with the percentage of correct target trials in inhibitory control (PCIT) by dividing the total number of correct target responses by the total possible target responses. Response time is calculated with the average response time for a correct target. For this study, each participant’s PCIT score during the 2- and 3-target trial on the second level was used in analyses separately as the increasing difficulty within three target levels does potentially measure other cognitive measures such as executive function.

### 2.3. Analyses

#### 2.3.1. Behavioral Impulsivity

We completed both group comparisons (no SIB history versus SIB history) and dimensional analyses (number of lifetime SIBs) with performance on behavioral measures of impulsivity. Lifetime SIBs were natural log transformed due to its highly positively skewed distribution. The main variable of interest for the BART performance included each participant’s average number of pumps for each trial in which the balloon did not pop (No Pop Mean Pumps; NPMP). For PGNG, we were interested in the percent of correct inhibitory trials for each participant (PCIT). Group comparisons consisted of adolescents with SIBs and without SIBs, controlling for age. For dimensional analyses, we used partial correlations for the performance variables of each BART (NPMP) and PGNG (PCIT) tasks, using the natural log-transformed number of lifetime SIBs and age as a covariate. Post hoc analyses were performed on the BART performance data and included an analysis of NPMP and PMP for first 10, second 10, and third 10 trials of the task to see whether the adolescent’s performance changed over time based on lifetime SIBs.

#### 2.3.2. Self-Report Impulsivity

Note: Analyses that investigated self-report impulsivity included a smaller sample size as some participants who completed the BART and PGNG did not complete the UPPS-P or were in study 3, which administered the TFI rather than the UPPS-P.

To evaluate whether our data are consistent with prior studies, we completed independent samples t-tests to determine significant differences in self-report measures of impulsivity between those with and without SIBs. We also calculated partial correlations to assess relations between the number of lifetime SIBs and self-report measures of impulsivity, controlling for age.

## 3. Results

### 3.1. Demographic Information

A total of 45 participants (13 male, 32 female) had at least completed self-report measures of impulsivity or rumination. Of these participants, 40 remitted MDD, 4 were in an active MDD episode, and 1 had no history of MDD, although that person did have SIBs and had a diagnosis of another depressive disorder. Thirty-one of 45 participants had engaged in suicidal or nonsuicidal self-injury in their lifetime. Further demographic information can be found in Table 2.

### 3.2. Behavioral Impulsivity

Fourteen adolescents without SIBs and 30 with SIBs had BART data. We used Levene’s test to evaluate the homogeneity of variance, which indicated heterogeneity in our t-test for group comparisons on NPMP (*p* = 0.027). In this case, we used a t-test with a Satterthwaite approximation for the degrees of freedom. Homogeneity of variance was not violated for the other t-tests. There were no significant differences in NPMP between groups with (*M* = 30.95, *SD* = 11.43) versus without lifetime SIBs (*M* = 35.34, *SD* = 7.42), *t*(37.27) = 1.53, *p* = 0.136. For PGNG, 13 adolescents without and 21 with SIBs had task data. There were no significant differences in PCIT for two target trials among individuals with (*M* = 0.81, *SD* = 0.21) and without lifetime SIBs (*M* = 0.70, *SD* = 0.27), *t*(33) = −1.25, *p* = 0.22 and no differences in PCIT for three target trials among individuals with (*M* = 0.61, *SD* = 0.19) and without lifetime SIBs (*M* = 0.59, *SD* = 0.26), *t*(33) = −0.234, *p* = 0.82.

**Figure 1:**
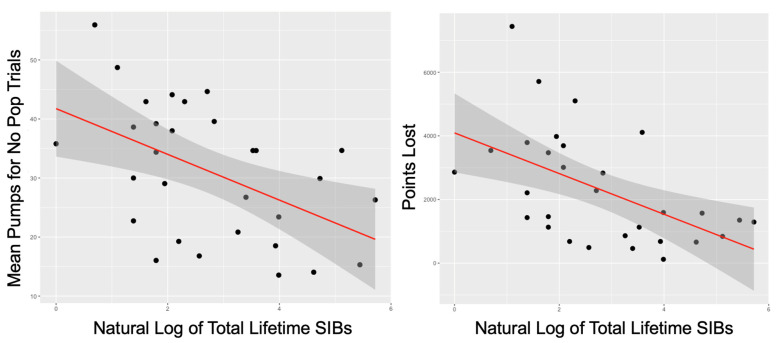
Relationships between SIBs and BART performance. **Left**: Negative correlation between natural log of lifetime SIBs and number of mean pumps for no pop trials on the BART. **Right**: Negative correlation between natural log of lifetime SIBs and number of points lost.

Dimensionally, the number of lifetime SIBs was negatively associated with the mean number of pumps for no pop trials (*r* = −0.50, *p* = 0.006) and number of points lost (*r* = −0.53, *p* = 0.003) but not with points won (*r* = −0.31, *p* = 0.12). This is shown in Figure 1. Number of lifetime SIBs was not associated with PGNG PCIT 2 target (*r* = 0.20, *p* = 0.291) or PCIT 3 target (*r* = 0.31, *p* = 0.103). There was no correlation between rumination with either BART or PGNG performance. Table 3 shows partial correlation results.

### 3.3. Self-Report Impulsivity and Rumination

Twenty adolescents with SIBs and 14 without SIBs completed self-report measures of impulsivity. Other adolescents from our sample that were not included either did not complete UPPS-P because they were enrolled prior to the addition of these measures or were from study 3, which used TFI rather than UPPS-P. Relative to adolescents with no SIB histories (*M* = 19, *SD* = 4.707), adolescents with lifetime SIBs (*M* = 22.65, *SD* = 4.308) showed significantly greater negative (but not positive) urgency as measured by the UPPS-P, *t*(32) = −2.341, *p* = 0.026.

When examining SIBs dimensionally (*n* = 19), there was a positive association between the natural log-transformed number of lifetime SIBS and both negative and positive urgency as measured by the UPPS-P (*r* = 0.516, *p* = 0.024 and *r* = 0.680, *p* < 0.001 respectively).

There were no significant differences between the SIB and nonSIB groups in total rumination (*t*(42) = 0.275, *p* = 0.785) or brooding rumination (*t*(41) = 0.294, *p* = 0.77). Within the whole sample, total rumination was associated with a lack of premeditation (*r* = 0.426, *p* = 0.013), negative urgency (*r* = 0.550, *p* < 0.001), and a lack of perseverance (*r* = 0.347, *p* = 0.048). The RRS brooding subscale was associated with a lack of premeditation (*r* = 0.473, *p* = 0.005) and negative urgency (*r* = 0.465, *p* = 0.006). There was no relation between SIBs and RRS total and RRS brooding (*r* = 0.374, *p* = 0.05 and *r* = 0.345, *p* = 0.078 respectively), although it is worth mentioning that these results are approaching significance. See Table 3 for partial correlation results.

### 3.4. Relations between Self-Report and Behavioral Impulsivity Measures

Given that studies investigating the relations between self-report and behavioral measures of impulsivity show inconclusive results, we aimed to clarify the relation by investigating varied measures of impulsivity and SIBs in our adolescent sample. Thirty-four participants completed both the UPPS-P and had BART task data. The only relation between the BART performance (NPMP and points lost) and self-report impulsivity was a negative correlation between NPMP and positive urgency (*r* = −0.539, *p* < 0.001). Thirty-one participants completed both the UPPS-P and PGNG, and there were no significant relations between any of the impulsivity indices on UPPS-P and PGNG performance.

**Figure 2:**
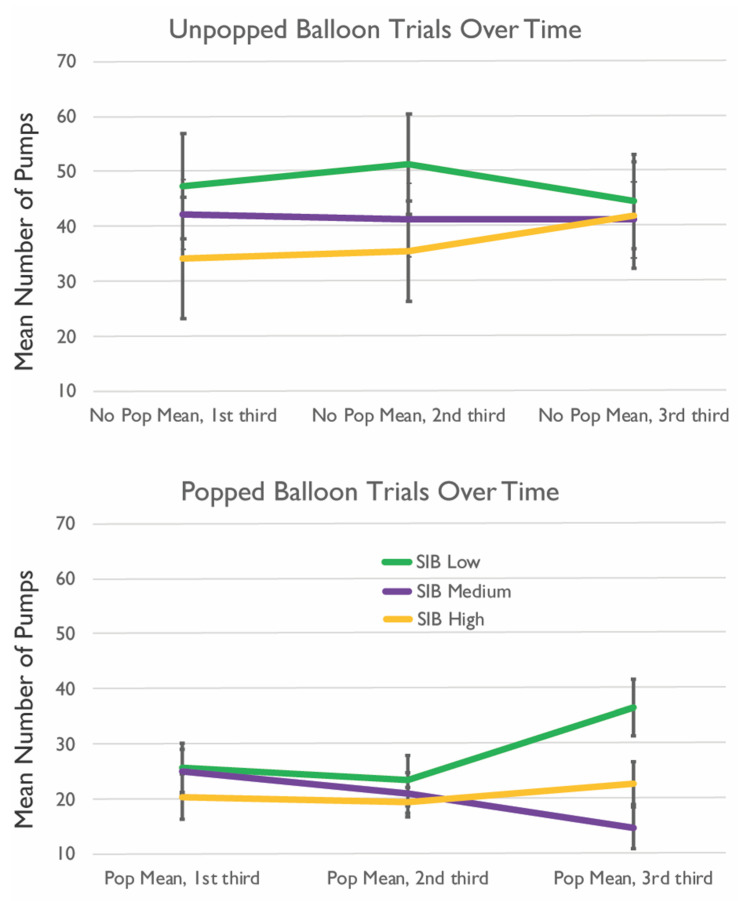
BART Performance over Time and SIB Frequency. (**Top**) Number of mean pumps for no pop trials across first, second, and third sections of BART by SIB frequency groupings. (**Bottom**) Number of mean pumps for trials in which the balloon popped across first, second, and third sections of BART by SIB frequency groupings.

**Figure 3:**
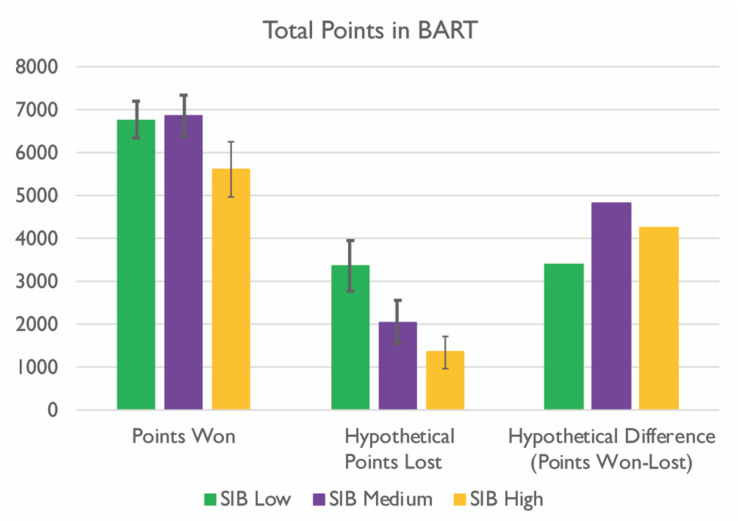
Points Won and Lost Across SIB Frequency Groups. Low, medium, and high frequency SIB groups showed different patterns of point wins and losses, with the high SIB frequency group winning and losing fewer points during the task.

To better visualize the relationships between NPMP on the BART and SIBs, we created figures by parsing the SIB group into low, medium, and high frequency and dividing the BART performance into the beginning, middle, and end of the task. We did this by dividing the sample into three groups of near-equivalent sizes based on the number of lifetime SIBs, yielding 10 participants for the low and medium SIB frequency groups and 7 for the high SIB frequency group. Low-frequency SIBs ranged from 1–7, medium from 8–34, and high from 35+ lifetime episodes of self-injury. We observed that the low-frequency SIB group, on average, had a mean number of pumps over the course of the task and for both no pop and pop trials. In contrast, the high-SIB group had, on average, lower mean pumps toward the beginning of the task and gradually increased by the last trials (Figure 2). A similar trend was seen when visualizing points won and lost as the high-SIB group won fewer points but also lost fewer points relative to the low-SIB group (Figure 3).

## 4. Discussion

Impulsivity is a challenging construct to examine when attempting to understand its relation to psychopathology. This has been especially demonstrated in its relation to SIBs, as some indices of impulsivity assessed via self-report have been reliably linked to SIBs, whereas behavioral measures of impulsivity have failed to consistently demonstrate such a relation [12,17]. Consistent with much of the previous research, our results demonstrated a significant relationship between SIBs and higher levels of the urgency dimension of impulsivity; this dimension measures an inner drive to do something, in this case, driven by strong positive or negative emotions. Further, we did not find significant relations with SIBs and a more commonly used measure of behavioral impulsivity, the PGNG task. In contrast, we did find that performance on the BART, a task focused on risk-taking, did indeed show a significant and unanticipated negative correlation with lifetime SIBs. A prior study of young adolescents (aged 10–13 years) similarly found a relation between the BART performance and SIBs [33].

The difference in performance between these measures can likely be attributed to the multifaceted nature of the construct of impulsivity. While the PGNG task examines motor inhibition, the BART evaluates risk-taking and impulsive choice. Further, PGNG does not typically use affect-laden stimuli, whereas the ability to earn and lose points on the BART has more potential to elicit some, albeit likely minimal, emotional response. The use of tasks that are more likely to evoke emotional experience are likely more ecologically valid, particularly when interested in behaviors that typically occur in the context of heightened emotions, such as SIBs. Future studies would also benefit from including brief self-report measures of their emotional experience immediately before or after these tasks.

Our study demonstrated that greater lifetime SIBs were associated with a lower number of mean pumps during the BART trials in which the balloon did not pop, indicating greater risk aversion. As SIBs are typically thought of as being associated with *greater* risk-taking, these results initially appear counterintuitive. However, there has been some evidence of greater risk aversion among individuals with SIBs [47]. Specifically, Baek and colleagues found that patients with depression and a history of suicidal behavior showed greater aversion toward loss during a monetary decision-making task, which they attributed to the tendency of these individuals to have negative biases in their predictions. Further, when compared to psychiatric patients with no suicidality, patients with suicidality showed a greater bias toward actively escaping aversive stimuli, consistent with the theory that suicidal behaviors are an effort to escape painful emotions [48]. The inclusion of risk and reward in the BART also highlights the difference in set-points for risk. It may be that SIBs are considered high control risks, and the BART is a low control risk situation. This could explain how high control teens with significant anxiety and mood problems are at greater risk for SIBs. This may require reconceptualizing nonsuicidal SIBs as a means to provide control via emotion regulation, whereas suicidal SIBs are a means to provide control through escape of aversive states.

An alternate explanation is that higher SIBs is associated with less sensitivity to reward. However, there is no significant linear association between lifetime SIBs and points won on the BART. Nonetheless, this will be worthy of further exploration in future studies with larger samples and possibly in the context of neuroimaging paradigms. Finally, another important consideration in understanding this relation is that most of the participants in our sample were currently experiencing a remission from MDD at the time of completing the task. While our small number of participants experiencing more acute psychopathology precluded post hoc analyses to verify this hypothesis, it is possible that for individuals who have engaged in SIBs at a higher frequency, overcompensation of risk avoidant behavior may be a necessary feature to achieve remission. Planned follow-up studies with this sample can differentiate whether this high control, avoidant subsample with more SIBs is on a trajectory toward sustained wellness, or if the phenotype is related to a shift in reward/risk balance that may portend missing important developmental milestones with some degree of risk (e.g., academic, occupational, and relational).

While not significant in our sample, the relationship between rumination and lifetime SIBs approached significance, with a greater number of SIBs being positively associated with rumination. As rumination is a passive process, it can contribute to avoidant behaviors. These avoidant behaviors may include risk aversion, such as that observed in the present study. Larger and more expansive studies are needed to evaluate this link and determine whether rumination may be a target of interest in reducing risk for SIBs.

A unique aspect of this study is the use of two different measures of behavioral impulsivity in addition to self-report which allowed for assessing possible convergence (or lack thereof) of these measures. Consistent with the literature, inhibitory control on the PGNG was not related to self-report impulsivity or SIBs. Important limitations of this study include its relatively small sample size. Further, our sample had a mixture of psychopathology, but the primary source for this sample was from a study recruiting adolescents with remitted MDD, many of whom had not engaged in SIBs for a relatively long period of time. To develop a better understanding of the relation between SIBs and impulsivity, greater care is needed to consider the role of active symptoms of psychopathology as well as recency of SIBs. This will require substantially larger samples of adolescents and should ideally incorporate multiple measures of both self-report and behavioral impulsivity given the multifaceted nature of the construct.

## 5. Conclusions

While highly preliminary given its sample size, the present study supports existing research demonstrating an inconclusive association between behavioral measures of impulsivity and SIBs, as our results differed across our two different behavioral measures. However, we did find that individuals with remitted MDD who have engaged in more frequent lifetime SIBs demonstrate greater risk aversion even when they may self-report higher levels of urgency. Because of the recovery status of these individuals and the link between SIBs and severe psychopathology, it is possible that this increased risk aversion is a necessary compensatory response to achieve remission. Future work will need to examine the role of acute versus remitted psychopathology and SIBs when considering impulsivity. Further, integration of other measures and methods (e.g., neuroimaging) will increase our understanding of the neurobiological mechanisms at play, thereby allowing for the implementation of neurobiologically-informed interventions to help reduce SIBs and related psychopathology.

## Figures and Tables

**Table 1:** Inclusion and exclusion criteria for studies 1, 2, and 3, which make up our present sample.

	Study 1	Study 2	Study 3	Present Sample
Inclusion Criteria	14–17 years Remitted MDD	14–17 years Active MDD	13–17 years SITBs in past month	Self-injurious behaviors (suicidal or nonsuicidal)
Exclusion Criteria	Suicidal ideation with plan and intent in past 6 months Psychotic disorders	Suicidal ideation with plan and intent in past 6 months Psychotic disorders	SITBs exclusive to psychotic episode	Pervasive developmental delay Autism spectrum disorder IQ < 80
Self-report and Interview Measures	KSADS-PL DSM5 LSASI UPPS-P RRS	KSADS-PL DSM5 LSASI UPPS-P RRS	MINI-Kid SITBI TFI RRS	MDD diagnosis SIB lifetime count UPPS-P

Major Depressive Disorder MDD; Self-Injurious (Thoughts and) Behaviors = SITBs; The Schedule for Affective Disorders and Schizophrenia for School Aged Children–Lifetime Version = KSADS-PL; Lifetime Suicide Attempt Self-Injury Count = LSASI; Self-Injurious Thoughts and Behaviors Interview = SITBI; Urgency, Premeditation (lack of), Perseverance (lack of), Sensation Seeking, Positive Urgency, and Impulsive Behaviors Scale = UPPS-P; Three Factor Impulsivity Index = TFI; Rumination Responsiveness Scale = RRS.

**Table 2:** Biological sex, Major Depressive Disorder Status, Race, and Ethnicity of the present sample.

General Demographics				
		SIBs (*n* = 31)	No SIBs (*n* = 14)	Total (*n* = 45)
Age	*M*(*SD*)	15.77 (1.09)	15.93 (1.00)	15.82 (1.05)
Sex	Male	9	4	13
	Female	22	10	32
Race	White	28	14	42
	Asian	1	0	1
	Native Hawaiian/Pacific Islander	1	0	1
	American Indian/Alaskan Native	1	0	1
	Total	31	14	45
Ethnicity	Hispanic	5	0	5
	Not Hispanic	26	14	40
	Total	31	14	45
Clinical Characteristics				
MDD Status	Remitted	26	14	40
	Active	4	0	4
	No MDD	1	0	1
	Total	31	14	45
Lifetime SIBs *M*(*SD*)	Raw Total	44.3 (72.27)	N/A	44.3 (72.27)
	Log Transformed	2.76 (1.46)	N/A	2.76 (1.46)
Self-Reported Impulsivity *M*(*SD*)	UPPS-P: Positive Urgency	18.9 (6.11)	17.43 (5.39)	18.29 (5.79)
	UPPS-P: Negative Urgency	22.65 (4.31)	19.00 (4.71)	21.15 (4.77)
Behavioral Impulsivity *M*(*SD*)	BART: NPMP	30.95 (11.43)	35.34 (7.42)	32.13 (10.42)
	PGNG: PCIT 2 Target	0.79 (.25)	0.70 (0.27)	0.76 (0.26)
	PGNG: PCIT 3 Target	0.61 (0.19)	0.59 (0.26)	0.60 (0.21)

SIB = Self-Injurious Behaviors; MDD = Major Depressive Disorder; UPPS-P = Urgency, Premeditation (lack of), Perseverance (lack of), Sensation Seeking, Positive Urgency, and Impulsive Behaviors Scale; BART = Balloon Analogue Response Task; NPMP = No Pop Mean Pumps; PGNG = Parametric Go/No-Go; PCIT = Percent Correct Inhibition Trials.

**Table 3:** Partial correlations.

	Lifetime Self-Injurious Behaviors (Natural Log Transformed)	Rumination
No Pop Mean Pumps	−0.498 **	−0.219
Points Lost	−0.532 **	−0.123
Points Won	−0.312	−0.190
2T PCIT	0.203	0.063
3T PCIT	0.291	−0.222
Negative Urgency	0.532 *	0.539 **
Positive Urgency	0.691 ***	0.285

* *p* < 0.05; ** *p* < 0.01; and *** *p* = 0.001. The correlations between Lifetime SIBs, Rumination Responsiveness Scale total score, Balloon Analogue Risk Task’s no pop pumps and points lost, and positive and negative urgency measured using the UPPS-P.

## Data Availability

The study is registered on clinicaltrials.gov (NCT03859297), which will beupdated with the published protocol and the study results and associatedpublications. De-identified data and results will be submitted to the NationalDatabase for Clinical Trials Related to Mental Illness (NDCT).

## References

[1] CDC WISQARS (Web-based Injury Statistics Query and Reporting System)|Injury Center|CDC. https://www.cdc.gov/injury/wisqars/index.html.

[2] Horwitz A.G., Czyz E.K., King C.A. (2015). Predicting Future Suicide Attempts Among Adolescent and Emerging Adult Psychiatric Emergency Patients. J. Clin. Child Adolesc. Psychol..

[3] Mars B., Heron J., Klonsky E.D., Moran P., O’connor R.C., Tilling K., Wilkinson P., Gunnell D. (2019). Predictors of future suicide attempt among adolescents with suicidal thoughts or non-suicidal self-harm: A population-based birth cohort study. Lancet Psychiatry.

[4] Klonsky E.D., May A. (2010). Rethinking Impulsivity in Suicide. Suicide Life-Threat. Behav..

[5] Carver C.S., Johnson S., Joormann J., Kim Y., Nam J.Y. (2011). Serotonin Transporter Polymorphism Interacts With Childhood Adversity to Predict Aspects of Impulsivity. Psychol. Sci..

[6] Carver C.S., Voie L.L., Kuhl J., Ganellen R.J. (2011). Cognitive Concomitants of Depression: A Further Ex-amination of the Roles of Generalization, High Standards, and Self-Criticism. J. Soc. Clin. Psychol..

[7] Cyders M.A., Smith G.T., Spillane N.S., Fischer S., Annus A.M., Peterson C. (2007). Integration of impulsivity and positive mood to predict risky behavior: Development and validation of a measure of positive urgency. Psychol. Assess..

[8] Whiteside S.P., Lynam D.R. (2001). The Five Factor Model and impulsivity: Using a structural model of personality to understand impulsivity. Pers. Individ. Differ..

[9] Patton J.H., Stanford M.S., Barratt E.S. (1995). Factor structure of the Barratt impulsiveness scale. J. Clin. Psychol..

[10] Steinberg L., Sharp C., Stanford M.S., Tharp A.T. (2013). New tricks for an old measure: The development of the Barratt Impulsiveness Scale–Brief (BIS-Brief). Psychol. Assess..

[11] Maxfield B.L., Pepper C.M. (2017). Impulsivity and Response Latency in Non-Suicidal Self-Injury: The Role of Negative Urgency in Emotion Regulation. Psychiatr. Q..

[12] Lockwood J., Daley D., Townsend E., Sayal K. (2017). Impulsivity and self-harm in adolescence: A systematic review. Eur. Child Adolesc. Psychiatry.

[13] Cassels M., Neufeld S., van Harmelen A.-L., Goodyer I., Wilkinson P. (2020). Prospective Pathways From Impulsivity to Non-Suicidal Self-Injury Among Youth. Arch. Suicide Res..

[14] Janis I.B., Nock M.K. (2009). Are self-injurers impulsive?: Results from two behavioral laboratory studies. Psychiatry Res..

[15] Furnham A., Henderson M. (1982). The good, the bad and the mad: Response bias in self-report measures. Pers. Individ. Differ..

[16] van de Mortel T.F. (2008). Faking It: Social Desirability Response Bias in Self-report Research|The Australian Journal of Advanced Nursing. Aust. J. Adv. Nurs..

[17] Hamza C.A., Willoughby T., Heffer T. (2015). Impulsivity and nonsuicidal self-injury: A review and meta-analysis. Clin. Psychol. Rev..

[18] Hedge C., Powell G., Bompas A., Sumner P. (2020). Self-reported impulsivity does not predict response caution. Pers. Individ. Differ..

[19] Alderson R.M., Rapport M.D., Kofler M. (2007). Attention-Deficit/Hyperactivity Disorder and Behavioral Inhibition: A Meta-Analytic Review of the Stop-signal Paradigm. J. Abnorm. Child Psychol..

[20] Bechara A., Damasio H., Tranel D., Damasio A. (2005). The Iowa Gambling Task and the somatic marker hypothesis: Some questions and answers. Trends Cogn. Sci..

[21] Lipszyc J., Schachar R. (2010). Inhibitory control and psychopathology: A meta-analysis of studies using the stop signal task. J. Int. Neuropsychol. Soc..

[22] Cyders M.A., Coskunpinar A. (2011). Measurement of constructs using self-report and behavioral lab tasks: Is there overlap in nomothetic span and construct representation for impulsivity?. Clin. Psychol. Rev..

[23] Mc Closkey M.S., Look A.E., Chen E.Y., Pajoumand G., Berman M.E. (2012). Nonsuicidal Self-Injury: Relationship to Behavioral and Self-Rating Measures of Impulsivity and Self-Aggression. Suicide Life-Threat. Behav..

[24] Gunten C.D., Bartholow B.D., Martins J.S. (2020). Inhibition Tasks are not Associated with a Variety of Behaviours in College Students. Eur. J. Pers..

[25] Allen K.J., Hooley J. (2015). Inhibitory control in people who self-injure: Evidence for impairment and enhancement. Psychiatry Res..

[26] Aklin W.M., Lejuez C., Zvolensky M.J., Kahler C.W., Gwadz M. (2005). Evaluation of behavioral measures of risk taking propensity with inner city adolescents. Behav. Res. Ther..

[27] Lejuez C.W., Read J.P., Kahler C.W., Richards J.B., Ramsey S.E., Stuart G.L., Strong D.R., Brown R.A. (2002). Evaluation of a behavioral measure of risk taking: The Balloon Analogue Risk Task (BART). J. Exp. Psychol. Appl..

[28] Broos N., Schmaal L., Wiskerke J., Kostelijk L., Lam T., Stoop N., Goudriaan A.E. (2012). The relationship between impulsive choice and impulsive action: A cross-species translational study. PLoS ONE.

[29] Weafer J., Baggott M.J., de Wit H. (2013). Test–retest reliability of behavioral measures of impulsive choice, impulsive action, and inattention. Exp. Clin. Psychopharmacol..

[30] Ellingson J.M., Potenza M.N., Pearlson G.D. (2018). Methodological factors as a potential source of discordance between self-report and behavioral measures of impulsivity and related constructs. Addict. Behav..

[31] Hunt M.K., Hopko D.R., Bare R., Lejuez C., Robinson E.V. (2005). Construct Validity of the Balloon Analog Risk Task (BART). Assessment.

[32] Lynam D.R., Miller J.D., Miller D.J., Bornovalova M.A., Lejuez C.W. (2011). Testing the relations between impulsivity-related traits, suicidality, and nonsuicidal self-injury: A test of the incremental validity of the UPPS model. Pers. Disord. Theory Res. Treat..

[33] Giannetta M.M., Betancourt L.M., Brodsky N.L., Wintersteen M.B., Romer D., Giannetta J.M., Hurt H. (2012). Suicidal Ideation and Self-Harm Behavior in a Community Sample of Preadolescent Youth: A Case-Control Study. J. Adolesc. Heal..

[34] Watkins E.R., Roberts H. (2020). Reflecting on rumination: Consequences, causes, mechanisms and treatment of rumination. Behav. Res. Ther..

[35] Nicolai K.A., Wielgus M.D., Mezulis A. (2016). Identifying Risk for Self-Harm: Rumination and Negative Affectivity in the Prospective Prediction of Nonsuicidal Self-Injury. Suicide Life-Threat. Behav..

[36] Selby E.A., Connell L.D., Joiner T.E. (2009). The Pernicious Blend of Rumination and Fearlessness in Non-Suicidal Self-Injury. Cogn. Ther. Res..

[37] Selby E.A., Franklin J., Carson-Wong A., Rizvi S.L. (2013). Emotional Cascades and Self-Injury: Investigating Instability of Rumination and Negative Emotion. J. Clin. Psychol..

[38] Miranda R., Nolen-Hoeksema S. (2007). Brooding and reflection: Rumination predicts suicidal ideation at 1-year follow-up in a community sample. Behav. Res. Ther..

[39] Rogers M.L., Joiner T.E. (2017). Rumination, Suicidal Ideation, and Suicide Attempts: A Meta-Analytic Review. Rev. Gen. Psychol..

[40] Buelens T., Luyckx K., Gandhi A., Kiekens G., Claes L. (2019). Non-Suicidal Self-Injury in Adolescence: Longitudinal Associations with Psychological Distress and Rumination. J. Abnorm. Child Psychol..

[41] Kaufman J., Birmaher B., Brent D., Rao U., Flynn C., Moreci P., Williamson D., Ryan N. (1997). Schedule for Affective Disorders and Schizophrenia for School-Age Children-Present and Lifetime Version (K-SADS-PL): Initial Reliability and Validity Data. J. Am. Acad. Child Adolesc. Psychiatry.

[42] Sheehan D.V., Sheehan K.H., Shytle R.D., Janavs J., Bannon Y., Rogers J.E., Milo K.M., Stock S.L., Wilkinson B. (2010). Reliability and Validity of the Mini International Neuropsychiatric Interview for Children and Adolescents (MINI-KID). J. Clin. Psychiatry.

[43] Linehan M.M., Comtois K.A. (1996). Lifetime Parasuicide History.

[44] Nock M.K., Holmberg E.B., Photos V.I., Michel B.D. (2007). Self-Injurious Thoughts and Behaviors Interview: Development, reliability, and validity in an adolescent sample. Psychol. Assess..

[45] Treynor W., Gonzalez R., Nolen-Hoeksema S. (2003). Rumination Reconsidered: A Psychometric Analysis. Cogn. Ther. Res..

[46] Langenecker S.A., Zubieta J.-K., Young E.A., Akil H., Nielson K. (2007). A task to manipulate attentional load, set-shifting, and inhibitory control: Convergent validity and test–retest reliability of the Parametric Go/No-Go Test. J. Clin. Exp. Neuropsychol..

[47] Baek K., Kwon J., Chae J.-H., Chung Y.A., Kralik J.D., Min J.-A., Huh H., Choi K.M., An C.Y., Lee N.-B. (2017). Heightened aversion to risk and loss in depressed patients with a suicide attempt history. Sci. Rep..

[48] Millner A.J., Ouden H.E.M.D., Gershman S.J., Glenn C.R., Kearns J.C., Bornstein A.M., Marx B.P., Keane T.M., Nock M.K. (2019). Suicidal thoughts and behaviors are associated with an increased decision-making bias for active responses to escape aversive states. J. Abnorm. Psychol..

